# Temporal Trends in the Secondary Metabolite Production of the Sponge *Aplysina aerophoba*

**DOI:** 10.3390/md10040677

**Published:** 2012-03-23

**Authors:** Oriol Sacristán-Soriano, Bernard Banaigs, Mikel A. Becerro

**Affiliations:** 1 Center for Advanced Studies of Blanes (CEAB-CSIC), Accés a la Cala St. Francesc 14, Blanes 17300, Girona, Spain; Email: osacristan@ceab.csic.es or osacriso7@gmail.com; 2 Environmental and Biomolecular Chemistry Laboratory, University of Perpignan, Via Domitia, 52 Paul Alduy Ave., Perpignan Cedex 66860, France; Email: banaigs@univ-perp.fr; 3 Natural Products and Agrobiology Institute (IPNA-CSIC), Avda. Astrofísico Francisco Sánchez 3, La Laguna, Tenerife 38206, Spain

**Keywords:** *Aplysina*, chemical defenses, chemical ecology, natural products, Porifera, satellite-derived SST, seasonality, temporal variation

## Abstract

Temporal changes in the production of secondary metabolites are far from being fully understood. Our study quantified, over a two-year period, the concentrations of brominated alkaloids in the ectosome and the choanosome of *Aplysina aerophoba*, and examined the temporal patterns of these natural products. Based on standard curves, we quantified the concentrations of aerophobin-2, aplysinamisin-1, and isofistularin-3: three of the four major peaks obtained through chemical profiling with high-performance liquid chromatography. Our results showed a striking variation in compound abundance between the outer and inner layers of the sponge. The ectosome showed high concentrations of bromocompounds during the summer months, while the choanosome followed no pattern. Additionally, we found that, from the outer layer of the sponge, aerophobin-2 and isofistularin-3 were significantly correlated with water temperature. The present study is one of the first to document quantitative seasonal variations in individual compounds over multiple years. Further studies will clarify the role of environmental, biological, and physiological factors in determining the seasonal patterns in the concentration of brominated alkaloids.

## 1. Introduction

Marine invertebrates are involved in a great variety of interactions, many of which are chemically mediated [1,2]. Not surprisingly, marine invertebrates are a potential source of natural, bioactive products that act against external threats [2,3]. These compounds often play multiple ecological roles, primarily protection against predators [4,5,6,7,8], competitors for space [9,10], biofoulers [11,12], or opportunistic pathogenic microorganisms [13,14]. 

Natural products have generated pharmaceutical and biotechnological interest due to their potential roles in human diseases [15]. However, novel drug development requires large amounts of the candidate species in screening for potentially bioactive compounds [16,17]. To that end, biological and ecological studies can provide guidelines for applied and biotechnological research. To collect sufficient amounts of bioactive compounds for testing, we need to know where the natural products are produced and stored (e.g., specific tissue or cells), what organisms are involved in their production (*i.e.*, the marine invertebrate, an associated microorganism, or multiple organisms), and what factors affect their production (e.g., biotic, abiotic factors). An important point to understand natural product production is whether or not secondary metabolites vary over time, behavior which is unknown for most species. Continuous quantitative data over multiple years will contribute significantly to understand their natural variability, providing additional information to contrast with proposed mechanisms of production and natural function.

Sponges are among the best-known producers of bioactive compounds [2,18,19]. Sponges are distributed in multiple, diverse habitats [12,20,21,22,23,24]; this property has generated an interest in sponges in the field of chemical ecology and in the pharmacognosy industry [25]. To date, research has focused mostly on novel drug discovery with highlights on the origin of natural products. In comparison, little is known about the temporal variability of the vast number of known bioactive compounds. Secondary metabolite production may vary as predicted by the optimal defense theory (ODT), outlined by McKey [26,27] and Rhoades [28], in plants, and tested by several authors [29,30]. The ODT assumes that the metabolic cost of secondary metabolite production for chemical defenses must meet the organisms’ needs and be balanced against the other terms in the energy budget (*i.e.*, reproduction and growth). This theoretical framework of terrestrial plant defenses is particularly relevant to sponge chemical ecology [31,32]. Variations in the abundance of natural products may respond to physical constraints such as hydrodynamism [33], depth [33,34,35], or water temperature [36]. Habitat [31], sponge size [31], competition for space [37], or against fouling [38] may also cause changes in sponge secondary chemistry. Many of these biotic or abiotic factors vary between months, seasons, and years [37,38,39] so the production of secondary metabolites may have strong temporal patterns.

The aim of the present study was to quantitatively assess the temporal variability of bioactive compounds in the demosponge *Aplysina aerophoba *(Nardo 1833)*.* This verongid is a rich source of brominated alkaloids (BAs), which are chemically well characterized [40,41,42,43]. BAs in this sponge are known to vary within the same specimen and at multiple geographic scales [44,45,46,47], but the temporal patterns of variation remain undescribed. Here, we aimed to determine whether the concentration of natural products varied at multiple temporal scales, from months, to seasons, and years. Our results showed that temporal changes in the concentration of BAs occurred in the outer layer (*i.e.*, ectosome) of the sponge while we did not detect any variation in the internal region (*i.e.*, choanosome).

## 2. Results and Discussion

### 2.1. Chemical Profile of *Aplysina aerophoba*

We quantified a total of 240 samples (2 sampling years × 12 months per year × 5 individual sponges per month × 2 tissues per specimen) to characterize the chemical profile. We identified four out of six major peaks: aerophobin-1 (Aero1; peak 3), aerophobin-2 (Aero2; peak 4), aplysinamisin-1 (Aply1; peak 5), and isofistularin-3 (Iso3; peak 6), by comparing their retention times and UV profiles to those of purified, characterized standard compounds. Of these, we finally quantified the three major BAs (Aero2, Aply1, and Iso3), because Aero1 was often under the detection limit in the HPLC analyses.

### 2.2. Natural Product Variations

**Figure 1:**
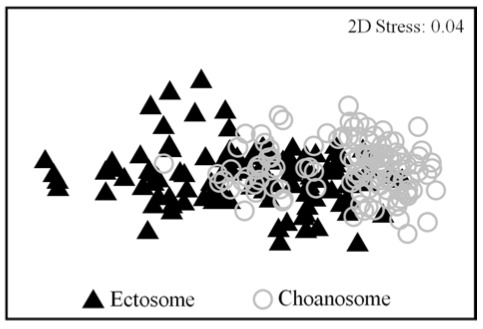
Metabolite abundances in the ectosome and choanosome of *Aplysina aerophoba*. Non-metric multidimensional scaling (MDS) was based on Bray-Curtis similarity matrices from standardized and square root-transformed abundances of chemical data. Significant differences were found between both layers.

The three compounds quantified showed significant differences in abundance in the ectosomal (outer) and choanosomal (inner) layers of the sponge (PERMANOVA, *P* = 0.001; Figure 1). Therefore, we subsequently performed separate analyses of the chemical data for these two layers. 

We found striking differences in the concentrations of secondary metabolites between the ectosome and the choanosome of *Aplysina aerophoba*. These differences were not only caused by changes in the abundance of the secondary metabolites but also in their dynamics. As reported for other sponges [37,48,49], *A. aerophoba* produces a heterogeneous mix of natural products. This heterogeneous mix of compounds is not uniformly distributed within the sponge. Based on X-ray energy dispersive microanalysis to locate bromine atoms [46], BAs in *A. aerophoba* were found in two sponge structures: the spherulous cells and the sponge fibers. Kreuter *et al.* [44] also detected intraspecimen differences in the amount of the low molecular weight (LMW) compounds, aeroplysinin-1 and dienone, between the external (*i.e.*, outer layer, oscular region) and the internal regions of *A. aerophoba*. These results were in agreement with a recent study [45] that found significant differences in the accumulation of high molecular weight (HMW) BAs between the choanosome (*i.e.*, inner region) and the ectosome (*i.e.*, outer layer) of the same species. The concentrations of the three compounds we quantified were positively correlated in both layers: ectosomal (Aero2-Aply1, *R* = 0.560; Aero2-Iso3, *R* = 0.658; Aply1-Iso3, *R* = 0.916; *P* < 0.001 for all comparisons) and choanosomal (Aero2-Aply1, *R* = 0.363; Aero2-Iso3, *R* = 0.571; Aply1-Iso3, *R* = 0.849; *P* < 0.001 for all comparisons). This could indicate that all compounds might be responding in the same way and at the same scale to the regulating factor(s).

The HMW BAs have been described as undergoing biotransformation when the tissue has been damaged, resulting in the LMW BAs aeroplysinin-1 and dienone, a process that is supposed to be enzyme-mediated [41,43]. The presence of these LMW BAs in our chromatograms could cast doubt on the actual concentration of the precursors quantified in our samples [42]. Yet, we never detected these bioconverted natural products in our chromatograms or they were at such low concentrations that cannot explain the large variation in BAs observed in our samples. This is expected, because our methods were designed to minimize the probability of biotransformation, keeping tissue manipulation to a minimum and avoiding the use of water. Moreover, Puyana *et al.* [50] found no evidence for biotransformation in two *Aplysina* species from the Caribbean. There is an unresolved controversy as to whether or not biotransformation occurs in *Aplysina* species and our study can neither support nor refute this biotransformation hypothesis. Our study was designed to investigate the temporal variation of the unaltered secondary chemistry of *Aplysina aerophoba*. If biotransformation does occur, our methods proved ideal to avoid biotransformation because we only found HMW BAs in our chromatograms, so biotransformation can neither affect nor explain the intraindividual variation observed in our study.

**Table 1:** *P*-values and percentage of the total variance explained by year, season, and month in the ectosome and the choanosome of the sponge. Values obtained from nested ANOVAs used to test the effect of timescale on the concentration of aerophobin-2 (Aero2), aplysinamisin-1 (Aply1), and isofistularin-3 (Iso3). We also show the percent variance unexplained by the factors (Error).

	Ectosome				Choanosome			
Compound	Year	Season	Month	Error	Year	Season	Month	Error
Aero2	0.869	0.190	0.027/14.85%	78.13%	0.059	0.491	0.030/13.67%	76.29%
Aply1	0.368	0.001/25.82%	0.539	74.18%	0.463	0.438	0.025/15.80%	83.80%
Iso3	0.260	0.003/24.44%	0.322	69.30%	0.551	0.201	0.031/14.03%	79.60%

**Figure 2:**
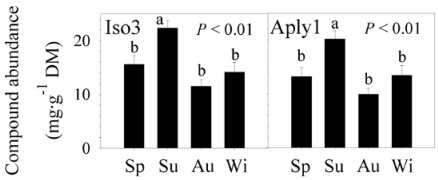
Seasonal changes in BA abundances. Secondary metabolite concentrations (mg·g^−1^ dry mass sponge tissue) ± 1 standard error of the mean (SE) observed in different seasons in the ectosome of* Aplysina aerophoba*. Sp = spring (*N* = 30); Su = summer (*N* = 30); Au = autumn (*N* = 30); Wi = winter (*N* = 30). Aply1 = aplysinamisin-1; Iso3 = isofistularin-3. Means (*i.e.*, seasons) with different letters are significantly different from each other (*P* ≤ 0.05; pairwise comparisons).

**Figure 3:**
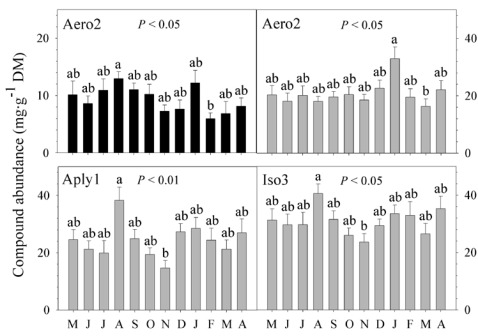
Monthly changes in metabolite concentrations. Secondary metabolite abundances (mg·g^−1^ dry mass sponge tissue ± 1 SE) in the ectosome (black) and in the choanosome (grey) of *Aplysina aerophoba* from May to April (*N* = 10 per month). We only show compounds in which there were significant monthly changes in metabolite concentrations. Aero2 = aerophobin-2; Aply1 = aplysinamisin-1; Iso3 = isofistularin-3. Means (*i.e.*, months) with different letters are significantly different from each other (*P *≤ 0.05; pairwise comparisons).

At the ectosomal level, there was no change in the abundances of the three compounds between years (nested ANOVA, *P* > 0.05 for the three BAs; Table 1). We found significant seasonal differences in the concentration of Aply1 and Iso3, with greater abundances in summer (nested ANOVA, *P* < 0.01 for both BAs; Figure 2, Table 1). However, there was no seasonal change in the abundance of Aero2 (nested ANOVA, *P* > 0.05; Table 1). Significant monthly differences were detected in the concentration of Aero2; the abundance increased in August and decreased in February (nested ANOVA, *P* < 0.05; Figure 3, Table 1). Nevertheless, the abundance of Aply1 and Iso3 did not vary between months (nested ANOVA, *P* > 0.05 for both; Table 1). Our results showed that “year” failed to explain the concentration of any of the three BAs investigated in the external layer of the sponge. However, “season” explained 25.82% and 24.44% of the total variance in Aply1 and Iso3 while “month” explained 14.85% of the total variance in Aero2 (Table 1).

At the choanosomal level, we found no annual differences in the abundances of the three compounds (nested ANOVA, *P* > 0.05 for the three BAs; Table 1). The concentration of the three BAs did not vary either between seasons (nested ANOVA, *P* > 0.05 for the three BAs; Table 1). However, we found significant monthly changes in the abundances of the three compounds (nested ANOVA, *P* < 0.05 for the three BAs; Figure 3, Table 1). Whereas the abundance of Aply1 and Iso3 increased in August and decreased in November, the concentration of Aero2 was higher in January and lower in March (Figure 3). Neither “year” nor “season” explained the concentration of the three BAs in the internal part of the sponge. But, “month” explained 13.67%, 15.80%, and 14.03% of the total variance in aerophobin-2, aplysinamisin-1, and isofistularin-3 (Table 1). 

### 2.3. Natural Product Trends

**Figure 4:**
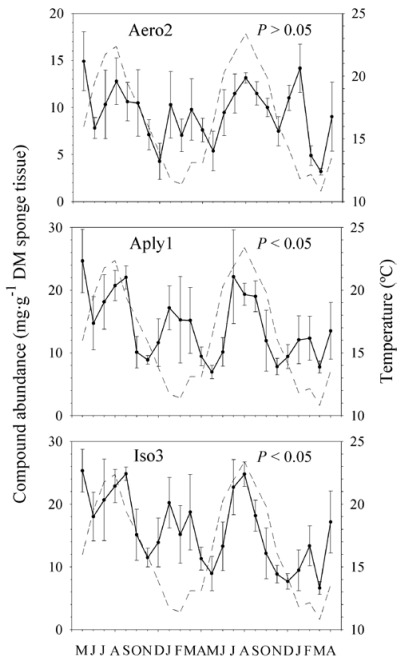
Temporal trends in metabolite abundances in the ectosome. Compound abundances (mg·g^−1^ dry mass sponge tissue ± 1 SE) observed in the ectosome of *Aplysina aerophoba* over a two-year survey (*N* = 5 per month). Aero2 = aerophobin-2; Aply1 = aplysinamisin-1; Iso3 = isofistularin-3. Significant differences *P *≤ 0.05. The sea surface temperatures (°C) are also shown for the whole period (discontinuous line).

**Figure 5:**
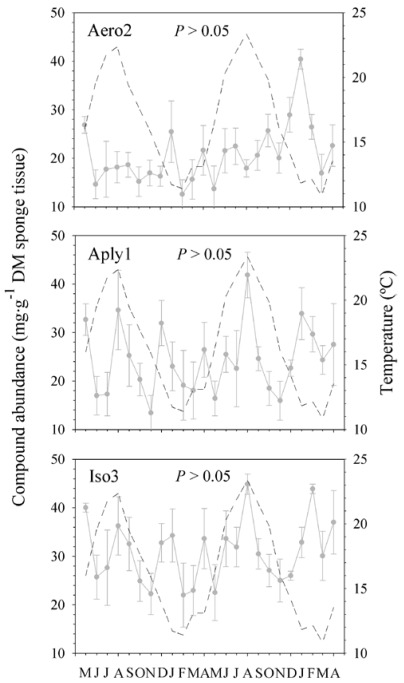
Temporal trends in metabolite abundances in the choanosome. Compound abundances (mg·g^−1^ dry mass sponge tissue ± 1 SE) observed in the choanosome of *Aplysina aerophoba* over a two-year survey (*N* = 5 per month). Aero2 = aerophobin-2; Aply1 = aplysinamisin-1; Iso3 = isofistularin-3. Significant differences *P *≤ 0.05. The sea surface temperatures (°C) are also shown for the whole period (discontinuous line).

Looking at the complete 2-year-long temporal series, we found in the external layer of the sponge significant changes in the concentration of Aply1 and Iso3 over time but not in Aero2 (*P* = 0.035, *P* = 0.038, and *P* = 0.711, respectively; Figure 4). In contrasting, we found no significant trend or seasonality in the abundance of BAs in the choanosome of the sponge (Aero2, *P* = 0.083; Aply1, *P* = 0.509; and Iso3, *P* = 0.566; Figure 5). 

Natural variations in sponge secondary chemistry have received little attention. An environmental influence on bioactive compound production was first documented by Thompson *et al.* [35] in the sponge *Rhopaloeides odorabile*. Seasonal patterns were reported later in several sponge species, where bioactivity was used to measure metabolite biosynthesis. Turon *et al.* [37] found *Crambe crambe* most biologically active in late summer and autumn. *Latrunculia* sp. nov. was most active in spring [38]. Swearingen & Pawlik [51] documented that *Chondrilla nucula* was most toxic in summer. In contrast, other sponge species, *Agelas oroides* and *Petrosia ficiformis*, appeared to be most bioactive during winter [34]. But, relatively few studies have described long-term, quantitative, temporal changes in individual biologically active compounds. 

We detected changes within but not between years in the BAs of *Aplysina aerophoba*. Our data showed that temporal variations in BAs were tissue-specific. The internal region (*i.e.*, choanosome) did not appear to follow a clear pattern of production, but changes in sponge secondary chemistry occurred at low temporal scale (*i.e.*, months). By contrasting, there seemed to be a clear trend in the external layer (*i.e.*, ectosome) with the highest production period in summer and a lower peak in winter. Whereas aerophobin-2 seemed to be more prone to variation between months, aplysinamisin-1 and isofistularin-3 varied significantly between seasons. In fact, we also showed evidence for an annual periodicity with greater abundances of these two compounds in the warmer season. Page *et al.* [33] reported a similar seasonal pattern in the concentration of two bioactive compounds of *Mycale hentscheli*, although they did not present evidence of an annual periodicity in secondary metabolite production. Similarly, Abdo *et al.* [36] documented that the concentration of one bioactive compound of *Haliclona* sp. was significantly higher in summer than in winter. 

Alternatively, our data could be the result of the chaotic nature of chemical variability. Our results could be supported by true variation in BAs, or could be the consequence of laboratory errors (as a result of our extraction methods and analyses). Experimental errors, however, could either amplify or reduce the true natural variation. Since our methods minimized compound alteration in this species and were consistent across all samples, we believe that our study accurately describes the natural temporal variation in the production of BAs in *A. aerophoba*. Although a notable percentage of the variation in BAs in *A. aerophoba *was explained by the temporal scales investigated in our study (up to 26%), most variance remained unexplained (Table 1). It is clear that other unknown factors account for such variability. Further analyses and understanding of the production of natural products will shed light on this area.

Variations in defense patterns among tissues can be explained by environmental or physiological constraints. We found a chemically rich sponge core surrounded by a less chemically rich tissue layer [45]. Traditionally, intraindividual differences in bioactive natural products were typically interpreted as evidence of the distinct roles of different tissues against predation, competition, or both [31,39,48,52]. In *Aplysina*, the BAs aerophobin-2, aplysinamisin-1, and isofistularin-3 seem to be anti-predatory deterrents [43,53]. 

From the perspective of the ODT, one would expect a more chemically enriched external layer due to its exposure to the environment. However, the higher concentration of BAs in the choanosome could suggest a sufficiently protected sponge in the external layer or low biological pressure from other marine organisms. Indeed, the nudibranch *Tylodina perversa* is the only known predator of *A. aerophoba* and preys preferentially on the ectosome of the sponge [54]. Thus, the higher internal abundance of BAs might minimize the effect of this specialist predator in the inner core of the sponge, similar to what has been described for other opisthobranch-sponge feeding interactions [49,55]. Concentrations of chemical defenses—up to 10% of sponge dry mass in the ectosome—might be sufficient to protect the sponge against generalist fish predators, as tested by Thoms *et al.* [56]. In our study, we found that surface concentrations of BAs varied between 0.3 and 2.6%, which may not be high enough to deter predators, as opposed to the chemically enriched inner core that might also act to effectively deter generalist predators [56]. However, the deterrent activity of BAs at such low concentrations should be tested. Fluctuations in the abundance of *T. perversa* could explain part of the variation found in BA production, but most of the ecological and biological data of this gastropod is unknown (e.g., abundance, annual cycle).

Other roles (*i.e.*, anti-fouling, competition, antibacterial) could also explain variations in secondary chemistry [11,12]. The higher bioactivity during the warmer period may have been a response to increased fouling on the sponge surface [38] or to an increase in competitors [37]. However, it seems unlikely that BA production was constrained by variations in the abundance of competitors or foulers, based on the anti-predatory role of the HMW BAs studied [43,53]. Indeed, only the LMW BA aeroplysinin-1 and dienone show strong antibiotic activity and may protect this sponge from invasion of bacterial pathogens [41,56,57] but these two compounds were absent or below threshold detection in our samples. These LMW BAs could however be readily available after biotransformation of the HMW BA precursors quantified in our study [41,42,43]. The possibility cannot be entirely ruled out that these biological constraints (*i.e.*, foulers, competitors, pathogens) vary over the year, causing seasonal changes in the biosynthesis of bioactive compounds. 

Seasonal changes in secondary chemistry may be also attributed to physiological factors related to differential investment in growth or reproduction. Leong and Pawlik [58] described a resource allocation trade-off between chemical defenses and growth in two *Aplysina* species, with maximum growth in summer months. The reproductive biology of verongid sponges is little known. *A. aerophoba* is thought to be oviparous and dioecious, common traits in the order Verongida [59,60,61]. This order displays a restricted gamete production period between May and July [59,62]. However, rope-form sponges (e.g., *Aplysina* species) appear to be adapted to undertake asexual reproduction by fragmentation; sexual reproduction is considered as a functional alternative with small investment in gamete production [60]. Data on growth and reproduction of *A. aerophoba* is scarce but it is likely to be similar to that of other verongid sponges. Taking into account data on other verongid species, maximum growth could coincide with maximum metabolite production in summer just after gamete production, suggesting that these sponges were not limited in energetic resources during the warmer season. However, there is no evidence to support this hypothesis and formal data on growth and reproduction would be required.

### 2.4. Sea Water Temperature and Concentration of BAs

We found no differences between the *in situ* and satellite-derived temperature data (paired *t*-test; *t* = 0.884, *df* = 10, *P* = 0.397) and the measurements were highly significantly correlated (*R* = 0.990, *P* < 0.001). Therefore, we used satellite-derived sea surface temperatures (SSTs) as a proxy for water temperature. 

**Figure 6:**
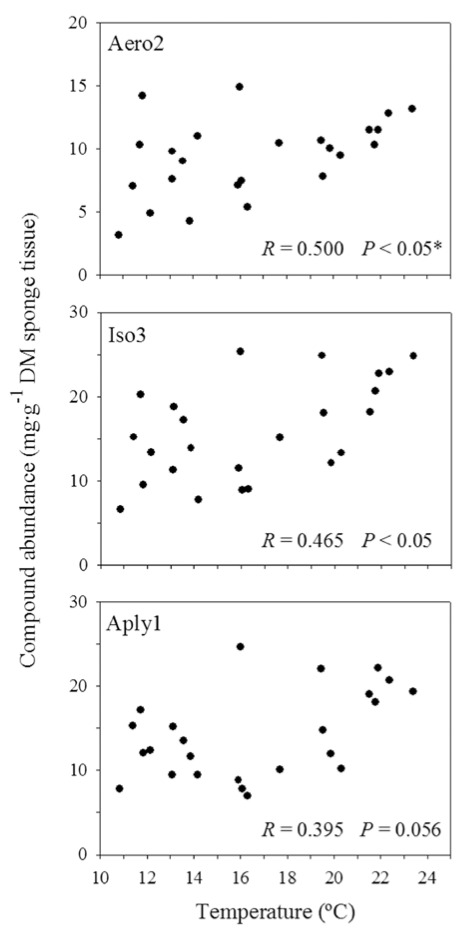
Correlations between compound abundances and satellite-derived sea surface temperatures. Changes in compound abundances (mg·g^−1^ dry mass sponge tissue) measured in the ectosome of *Aplysina aerophoba* were related to changes in sea surface temperature (°C). Aero2 = aerophobin-2; Aply1 = aplysinamisin-1; Iso3 = isofistularin-3. Significant differences *P* ≤ 0.05. * Significant correlation after Bonferroni correction.

At the ectosomal level, the abundances of Aero2 and Iso3 were positively correlated with SST (*R* = 0.500, *P* = 0.013; and *R* = 0.465, *P* = 0.022, respectively; Figure 6). If any, Aply1 was marginally correlated with the satellite-derived SST (*R* = 0.395, *P* = 0.056). Contrastingly, the abundances of Aero2, Aply1, and Iso3 in the choanosome were not correlated with SST (*R* = −0.079, *P* = 0.713; *R* = −0.009, *P* = 0.968; and *R* = 0.086, *P* = 0.689, respectively).

Variations in chemical defenses may be also attributed to environmental factors, such as water temperature. We showed that satellite-derived SST data could be accurately used as a proxy for sea water temperature pattern, consistent with other reports [63,64]. Some BAs were significantly correlated with sea water temperature in the external part of *Aplysina aerophoba*. Water temperature could be directly affecting the secondary metabolite production as reported in *Haliclona* sp. [36] or other factors (*i.e.*, biological or physiological) correlated with sea water temperature might be causing such variation [31,33,34,35,37,38]. In our study there appeared to be other factors that regulate the production of BAs. We based this hypothesis on the low correlation strength (*R* value ≤ 0.5) and the partial mismatch between temperature and ectosome patterns over time (Figure 4). The relationship between BAs and temperature could imply no causation. In our opinion, water temperature does not cause changes in secondary chemistry, since those changes should then occur both in the ectosome and choanosome of the sponge. Other environmental factors (e.g., light exposure) could differentially affect the surface layer but not the inner region of the sponge [33,35] and could be driving these trends. Biotic factors that also preferentially affect the ectosome of the sponge and are associated with temperature could be behind the increased compound concentration of the ectosome in summer [39]. It is known that photosynthetic symbionts can contribute to the production of secondary metabolites [65,66,67]. Since most photosymbionts are found in the outer layer of the sponge, these associated bacteria could be driving somehow the secondary chemistry of *A. aerophoba* [45,68]. Although the BAs seem to be stored in sponge cells [46], multiple cell components might be involved in their production [41,44,45,68]. The actual role of symbionts in the production of secondary metabolites, however, is largely unknown.

## 3. Experimental Section

From May 2008 to April 2010, five different healthy specimens of the sponge *Aplysina aerophoba* (Nardo 1833) were sampled monthly by scuba diving in Portbou (Northwestern Mediterranean) to depths between 7 and 10 m. Sampling was always conducted on fresh individuals with similar sizes, which were separated from each other between 2 and 100 m. Underwater, we cut off the upper part of one chimney per specimen and placed them in plastic bags with sea water. Immediately after collection, samples were placed in coolers with ice to prevent changes in the secondary chemistry. Once in the laboratory, a small portion of the top half of the chimney away from the cutting surface (*i.e.*, injured tissue) was selected for the quantification of the BAs, minimizing a potential effect of manipulation and the biotransformation of high molecular weight metabolites into low molecular weight compounds [41]. We used a sterilized scalpel to excise samples from the ectosome (1 mm-thickness) and choanosome (2 mm-thickness) of the sponge under sea water. Samples were then frozen at −20 °C within 3 hours of collection until processing. 

The major brominated alkaloids (BAs) of *Aplysina aerophoba* were extracted, isolated and identified as described by Sacristán-Soriano *et al.* [45]. Briefly, we reduced sponge manipulation, avoided air exposure of fresh samples, froze sponges immediately after sample preparation (less than 4 hours after collection), freeze-dried material and keep them at −20 °C, and used methanol only for extraction. With this methodology, we have never detected the biotransformation process that converts the High Molecular Weight (HMW) BAs into Low Molecular Weight (LMW) BAs described for this species. LMW BAs were either absent in our chromatograms or at concentrations below our detection threshold. We purified the four major and known compounds aerophobin-1, aerophobin-2, aplysinamisin-1, and isofistularin-3 as described by Sacristán-Soriano *et al.* [45]. We characterized these compounds with proton and carbon nuclear magnetic resonance (^1^H and ^13^C NMR; JEOL EX 400 spectrometer), liquid chromatography-mass spectrometry (LC-MS; Thermo Scientific LCQ Fleet), UV spectrometry (Hewlett Packard diode array spectrophotometer), and comparison of spectroscopy data with published values from the literature [40,69]. Full details on the chemical methods can be found in Sacristán-Soriano *et al.* [45].

HPLC analyses were performed with a system from Waters that included the Alliance separations module 2695, the column heater, and the 2998 photodiode array detector. The equipment was controlled and the data were handled by the Empower Chromatography Data software (Waters). The HPLC conditions consisted of two eluents (A: 0.1% aqueous trifluoroacetic acid, B: acetonitrile) and an elution profile based on a linear gradient from 30% B to 80% B within 18 min and then to 100% B in an additional 10 min. The flow rate was constant at 0.4 mL·min^−1^. We used a Phenomenex Synergi Max-RP (80 Å, 250 × 3.0 mm, 4 µm) analytical column with a fixed temperature of 30 °C.

For quantification of the natural products, 30 mg of freeze-dried sponge tissue from ectosomal and choanosomal samples were prepared by extracting three times with 1.5 ml of methanol (MeOH) in an ultrasonic tank for 15 min [45]. The crude extract was filtered through a 20-μm polytetrafluoroethylene filter (PTFE) into a 5 mL beaker. The final volume was adjusted to 5 ml of crude extract. Aliquots of 1.5 ml were passed through a 13 mm, 0.2-μm PTFE syringe-filter before injecting 10 μL into the HPLC system. The brominated compounds were detected at 245 nm from data collected across the 210–800 nm wavelength range. Peak areas were integrated and quantified with calibration curves based on previously purified and characterized external standard compounds. The final amounts of the natural compounds were calculated by averaging three replicate injections. Concentrations of BAs were expressed in mg·g^−1^ of dry mass of sponge tissue.

We obtained sea surface temperatures (SSTs) at the study site throughout the 2-year study (May 2008 to April 2010) by accessing moderate-resolution imaging spectrometer measurements (Aqua MODIS satellite) made by the NASA Goddard Space Flight Center [70]. “Ocean Level-2” HDF data were read and processed with MathLab R2009a software. High-quality readings were obtained with flag values of 0 or 1 and we discarded unreliable data (flag values of 2 or 3). We used a flag value of 1 because insufficient readings were obtained with a flag of 0. Suitable SST readings (*N* = 293) corresponded to daily means in a 9-km^2^ area centered at the following coordinates: 42°25'33.6"N-3°12'40.32"E (Portbou). We also measured sea water temperatures with *in situ* data loggers (Onset Computer Corporation, model HOBO Pendant UA-002-08) placed at a depth of 7 m for one year (June 2009 to April 2010) for comparisons with satellite-derived SST data. Monthly average temperatures were calculated for both satellite-derived and logger-derived data.

We used PRIMER 6 software [71] to analyze data on secondary metabolites of *Aplysina aerophoba* as a function of tissue type (ectosome or choanosome). Square root-transformed data were used to calculate Bray-Curtis similarities. We used permutational multivariate analysis of variance (PERMANOVA) to test for differences in secondary metabolites among different tissue layers. Results were plotted with non-metric multidimensional scaling (MDS).

We used several statistical methods from SYSTAT 12 software [72,73] to analyze each secondary metabolite separately. We used a nested design of ranked compound abundances with year (first and second), season (spring, summer, autumn, and winter), and month (12 months) as factors. Seasons were defined by natural periods (not by temperature) so they contained the same months in both years. Nested designs require random subordinate groups. We treated month and season as random factors because their actual conditions vary tremendously between years, *i.e.*, despite sharing the same name, months and seasons could be confined to their specific years. In nested analyses, lower variables are always tested before upper variables. To test for the significance of an upper level, the model considers the variance associated with the lower level, so there may be differences in an upper level regardless the significance of the levels below. With this hierarchical approach, we can therefore partition the variance into components associated with each temporal scale (year, season within years, and months within seasons) to assess the temporal scale at which concentration of BAs vary the most. This approach can provide hints on the mechanisms behind the temporal pattern. We then used Tukey HSD pairwise comparisons to test for differences between the levels of each significant temporal scale. We used simple correlation analysis to establish the quantitative relationships between secondary metabolites. We also performed trend analyses of compound data to assess for tendencies and seasonal variations over time in the abundances of secondary metabolites.

Monthly logger and satellite-derived temperature data from the same period were compared with paired t-tests to identify differences between methodologies. Pearson’s correlation was used to examine associations between ranked compound abundances and satellite-derived SSTs.

## 4. Conclusions

To date, the present study was one of the first to document long-term (two years) quantitative seasonal variations in bioactive secondary metabolites in sponges. We provided evidence for high compound accumulation during summer months in the verongid *Aplysina aerophoba*, as previously described for other sponges. This extended our understanding of the underlying patterns of production of biologically active metabolites. Beyond the biological and ecological consequences, being aware of such variation can contribute to a sustainable wild harvest or aquaculture supply for biotechnological research [16], minimizing the extraction of sponge biomass. For example, our data could cause a two-fold increase in the supply efficiency of aplysinamisin-1 or isofistularin-3, should they be targets of biotechnological research or commercial applications. So, just by harvesting sponges at times of peak metabolite concentration we could reduce by up to 50% our impact in natural populations and our expenses to extract the target natural product.
